# Supplementary material for: Calibrating the Human Mutation Rate via Ancestral Recombination Density in Diploid Genomes
Source: PLoS Genet. 2015 Nov 12;11(11):e1005550. doi: 10.1371/journal.pgen.1005550 (PMC4642934; doi:10.1371/journal.pgen.1005550)
Supplement: S1 Text — Additional technical details related to population size history inference, windows of measurement for correction factors, genotype error, gene conversion, base content, mutation rate heterogeneity, population heterogeneity & admixture, natural selection, and symmetry of uncertainty. (PDF) [file pgen.1005550.s001.pdf]

S1 Text: Supplementary Methods

Technical details for population size history inference

PSMC runs on a reduced version of the genome, with consecutive sites grouped into bins of 100 and each bin marked as 1 or 0 depending on whether there is at least one heterozygous site in the bin or not. Bins can also be marked as "missing" if a certain number of the 100 sites have un-called genotypes (90 in the original PSMC publication). We find that two aspects of this procedure can affect the overall average heterozygosity of simulated data generated from a PSMC-estimated population size history. First, different values of the missing-bin threshold lead to different heterozygosity levels. Second, the PSMC program accepts a maximum of one heterozygous site per bin, whereas there can in fact be more than one. This effect is non-linear as a function of TMRCA; since heterozygosity varies substantially along the genome, the program will systematically underestimate the age of the most anciently coalesced regions.

To account for these factors, we first use an empirically-determined threshold of 35 un-called sites per 100 to call a bin as missing, which yields a more closely matching final heterozygosity. Second, we implement a multiple-het-per-bin adjustment, whereby we modify the PSMC output before using it to create the calibration data, as follows. The population size history inferred from PSMC consists of discretized time intervals $[t_i \ t_{i+1}]$ (in coalescent units), with the population size s_i in each interval telling us how much of the genome falls within that level of TMRCA (and hence of per-bin heterozygosity). Given these heterozygosity levels, we use a binomial distribution to scale the times defining the endpoints from the output units of probability of at least one heterozygous site per 100 bp to the implied expected number of heterozygous sites per 100 bp. Creating calibration data according to these new values more accurately recapitulates the true distribution of

heterozygosity levels across the genome, as well as the total genome-wide heterozygosity.

Explicitly, if θ is the original baseline PSMC-inferred `ms` diversity parameter (per base), then we define the adjusted times T_i (with $T_0 = 0$) and sizes S_i recursively as

$$T_i = T_{i-1} + 2((1 - (1 - 100\theta * (t_i + t_{i-1}))^{0.01})/2\theta - t_{i-1})$$

and

$$S_i = s_i * (T_i - T_{i-1})/(t_i - t_{i-1}).$$

As an example, for our eight-genome PSMC inference, the population size trajectory (in `ms` notation) is adjusted from

```
-eN 0.0049 0.1053 -eN 0.0080 0.0562 -eN 0.0118 0.0653 -eN 0.0162 0.1052 -eN 0.0214
0.1860 -eN 0.0277 0.3115 -eN 0.0350 0.4524 -eN 0.0438 0.5549 -eN 0.0542 0.5847
-eN 0.0664 0.5498 -eN 0.0810 0.4846 -eN 0.0983 0.4185 -eN 0.1188 0.3646 -eN 0.1431
0.3257 -eN 0.1718 0.3006 -eN 0.2060 0.2882 -eN 0.2464 0.2880 -eN 0.2944 0.3003
-eN 0.3513 0.3275 -eN 0.4187 0.3726 -eN 0.4987 0.4363 -eN 0.5935 0.5102 -eN 0.7059
0.5755 -eN 0.8391 0.6142 -eN 0.9971 0.6254 -eN 1.1844 0.6360
```

to

```
-eN 0.0049 0.1053 -eN 0.0080 0.0564 -eN 0.0118 0.0655 -eN 0.0163 0.1060 -eN 0.0215
0.1874 -eN 0.0279 0.3152 -eN 0.0353 0.4587 -eN 0.0442 0.5653 -eN 0.0549 0.5978
-eN 0.0674 0.5655 -eN 0.0825 0.5012 -eN 0.1006 0.4364 -eN 0.1221 0.3834 -eN 0.1480
0.3464 -eN 0.1789 0.3237 -eN 0.2163 0.3153 -eN 0.2613 0.3210 -eN 0.3161 0.3424
-eN 0.3827 0.3839 -eN 0.4645 0.4517 -eN 0.5655 0.5513 -eN 0.6917 0.6788 -eN 0.8512
0.8168 -eN 1.0565 0.9467 -eN 1.3276 1.0732 -eN 1.6989 1.2608
```

As can be seen, the correction is negligible for more recent times where the density of heterozygous sites is low (and where our test regions have their TMRCA) and only becomes substantial for the oldest time intervals.

Windows of measurement for correction factors

As discussed in Methods ("Genotype error"), because our method works by calibrating the mutation rate against the recombination rate via the local heterozygosity and non-recombined tract length at our starting points (equivalently, the intercept and slope of $H_S(d)$), this local behavior is what we are concerned with when correcting for genotype error, gene conversion, and base content. These processes affect the entire $H_S(d)$ curve, but the calibration data recapitulate the full patterns of heterozygosity in the real data through the PSMC inference. Thus, once we fit the real-data curve to the calibration curves, which are ascertained to have similar $H_S(0)$, the question becomes how much this value $H_S(0)$ is impacted by various processes. While we would ideally like to derive the correction parameters directly at the starting points, in practice, we need to define windows around these points in order to obtain stable counts (as in the case of determining local heterozygosity in the initial ascertainment step). Aiming for a balance between capturing the behavior at the midpoint and maintaining high enough statistical power, we use windows of 10 kb when measuring the recombination rate for the gene conversion correction, 30 kb when measuring GC content and CpG site fraction for the base content mutability adjustment, and 50 kb when measuring CpG transition proportion for the genotype error correction (see below for more details for all three).

Details of genotype error rate estimation

To estimate the genotype error rate, we take advantage of the fact that C-to-T transitions at CpG dinucleotides are greatly enriched among true mutations as compared to genotype errors. We assume that genotype errors are equally likely at CpG and non-CpG sites; while this may not strictly be true, the frequency of CpG sites in the genome is low

enough (less than 2%) that deviations should only have a minor impact. More generally, we assume that the *a priori* probability of observing a genotype error is uniform across the genome. Because errors are rare, we do not have power to detect local variation in their rate of occurrence, and instead we aggregate over regions to produce as accurate an average error rate as possible.

For the fraction of CpG transitions among true heterozygous sites, we use a previous estimate of approximately 17.3% based on observation of almost 5000 *de novo* mutations [8]. Since GC-biased gene conversion results in a decrease in this frequency among older variants [3, 44], we use our gene conversion adjustment from above (roughly 0.14×10^{-8} per base per generation, with an order of magnitude more mutations introduced than removed for haplotypes in our TMRCA range), together with an estimated 2:1 transmission bias for GC bases [21], to estimate a reduction of approximately 0.6% for CpG transition frequency for our test regions. Given that our test regions have TMRCA roughly 10% of genome-wide average, this value is in line with previous reports of the difference in CpG transition fraction among *de novo* mutations versus common polymorphisms [3, 44]. To account for uncertainty, we use a standard error of 0.1% on the gene conversion correction (see below).

Using the same eight genomes as for our final μ estimates, with the same filtering and ascertainment of starting points, we compile the fraction of CpG transitions out of all heterozygous sites in the regions with local heterozygosity rates of 5–10 per 100 kb, with one caveat. Because CpG fraction strongly depends on the proportion of CpG sites, and we find that our test regions have relatively low such proportions (where we define CpG sites based on the human reference sequence), we truncate the low end of this distribution and only use test regions with at least 1.24% CpG sites (corresponding to 3551, or 37%, of the 9626 test regions), such that the overall proportion of CpG sites equals 1.85%, the

same as in [8]. Additionally, we only use the central half of the 100 kb regions (i.e., 25 kb on either side of the starting points) to represent more precisely the local error rate at the starting points (while maintaining adequate statistical power). We then compare the observed CpG fraction (about 14.4%) to the gene conversion-adjusted expectation for true mutations (about 16.7%), from which we infer the presence of genotype errors at a rate of 1.08×10^{-5} per base (roughly 1 per 100 kb, or one-seventh of the heterozygous sites in our test regions). We note that the GC content in the thresholded regions is approximately 42% on average, which would imply a slight decrease in CpG mutability as compared to the genome-wide average (see base content adjustment below), but to be conservative (i.e., err on the side of slightly over-correcting), we do not make this adjustment here.

As described in Methods, we apply our correction by multiplying the raw inferred μ values by $(H_S(0) - \epsilon)/H_S(0)$, where ϵ is the error rate. We incorporate this factor separately for each jackknife replicate, but using the same value of ϵ throughout, as we found that re-computing the error rate within the jackknife was unstable. Instead, we account for our noisy estimate of the error rate by translating our uncertainty in ϵ into uncertainty in μ (see Methods). We derive the former by sampling 100,000 random iterations of the three parameters of our error model: the true proportion of CpG mutations (from a binomial distribution with $n = 4933$ and $p = 855/4933$, recapitulating the counts from [8]), the proportion of CpG mutations in our data (likewise, with counts of 1165 out of 8108), and the gene conversion adjustment (see above). Taking the mean and standard deviation of this set, we obtain our estimate of $\epsilon = 1.08 \pm 0.28 \times 10^{-5}$. Finally, because of the relatively uniform preparation of our data, the reasonably large standard error, and our limited power to detect statistically significant differences, we use this same error rate for all samples.

Details of gene conversion correction

As discussed in Methods, the magnitude of the gene conversion effect depends on both the prevalence of non-crossover gene conversion events and the rate at which they introduce heterozygous sites. We make use of a recent direct estimate that non-crossover gene conversion affects 5.9×10^{-6} bases per generation [21], which is the product of the rate of events and their average length (on the order of 100 bases in humans [21, 43]). This rate is a genome-wide value, however, and the frequency of gene conversion is highly variable and correlated with local crossover rate [21, 45]. We assume that the gene conversion rate is proportional to the local recombination rate in our genetic map. Because of the way our starting points are chosen, they tend to lie in the relatively recombination-poor regions between hotspots, and so for each of our estimates, we multiply the average 5.9×10^{-6} gene conversion rate by the ratio of the local recombination rate (measured in 10 kb windows around the starting points) to the genome-wide average (1.19 cM/Mb, as measured in the deCODE map [16], which was used in [21]). As an example, for our primary eight-genome $H_{5\text{–}10}(d)$ curve, the starting-point windows cover 96 Mb but only 51 cM, less than half the average. We note that the differences in average recombination rate per base are only about 10% between the full 100 kb test regions and the central 10 kb windows and 25% or less between the central windows and the full super-regions (S2 Table). To capture noise in the measurement of the local rate, we calculate it separately for each replicate in the jackknife.

The second component of the gene conversion effect is the probability that a gene conversion event introduces a polymorphism that we observe as a heterozygous site. A very simple model for this rate would be that it equals the probability that a randomly chosen base differs from the homologous base on another chromosome in the population (from which it might have been copied in the event of a gene conversion at some point

in the past). This quantity can be estimated simply as the heterozygosity in a diploid genome. To this basic model, we add two additional details. First, although our test regions have relatively low heterozygosity (i.e., are relatively recently coalesced), there is also a chance that new mutations that have accumulated on either haplotype could be replaced by the ancestral base via gene conversion. For this probability, we use the heterozygosity at our starting points, $H_S(0)$, divided by 2 (because the new mutations have increased in number since the TMRCA). Second, the probability of mismatch with another chromosome could change over time, and we also find that because of base content, correlated coalescent histories, or other factors, the heterozygosity in other genomes is reduced around our ascertained starting points. For example, other European and East Asian genomes have heterozygosity 5.5–5.9 $\times 10^{-4}$ in the 5–10 het regions defined in French B, roughly 15–25% below the genome-wide average (see S1 Table). We assume an average value of 5.7×10^{-4} both for European test genomes (which have the highest heterozygosity) and other populations, with the exception of Karitiana, for which we instead use their total genome-wide heterozygosity (5.4×10^{-4}).

Finally, we assign an uncertainty to our correction, which is incorporated into the standard errors we report for our real-data mutation rate estimates. For the rate of gene conversion, we translate the estimated 95% confidence interval of 4.6–7.4 $\times 10^{-6}$ per base per generation [21] into a standard deviation of 0.7×10^{-6}, and for the probability of introducing a new polymorphism, we use a standard deviation of 10% of the total (i.e., $5.7 \pm 0.57 \times 10^{-4}$ for most applications).

To test the validity of this approach, we run simulations in which the test data are generated with gene conversion active, and the rest of the procedure is carried out as normal, including the correction just described. For this scenario, we use a true mutation rate of 1.5×10^{-8} per base per generation, and for computational efficiency, we assume

a uniform rate of gene conversion with $f = 3.75$ (i.e., 3.75 times the average crossover rate) and an average tract length of 100 bases. This results in a gene conversion rate of approximately 4.3×10^{-6}—about three-quarters of the true rate in humans, but roughly 50% higher than expected for our real-data starting points because of the correlation with local recombination rate. When applying the final correction, we use the average heterozygosity over all simulated genomic segments (approximately 6.6×10^{-4}) as the "donor" polymorphism probability.

Details of base content mutability adjustment

Our base content adjustment consists of three elements, which we use to form a ratio of the genome-wide mutation rate to the average local per-base mutation rate around our starting points. First, we compare the fraction of CpG sites between our starting-point regions and the full genome (1.58% after filtering) and use the relative mutation rates from [8], with uncertainty allowed for sampling noise (ratio of $\mu_{\mathrm{CpG}}/\mu_{\mathrm{other}} = 11.2 \pm 0.6$). Similarly, we compare total GC content (40% genome-wide), where non-CpG GC bases are assumed to be 50% more mutable than AT bases [44] (including uncertainty, 50±15%). Statistics for our test regions can be found in S2 Table.

Finally, we adjust for the fact that CpG sites are less mutable in regions of high GC content, due to a combination of strand separation effects and low methylation in CpG islands. We modified a result from [46], who estimated an exponential dependence of CpG deamination on local GC content. We (a) used their full CpG transition rates; (b) omitted the tails of the distribution by restricting to GC content between 30% and 60%; and (c) fit a linear dependence instead, both because we are using a genome-wide average and because a linear function appeared to be more accurate in this parameter range. A good fit was provided by the equation $\mu_{\mathrm{rel}} = 1 - 2.5\Delta_{\mathrm{GC}}$, where μ_{rel} is the CpG mutation rate

as a proportion of the average (at genome-average GC content of 40%), and Δ_{GC} is the local GC content fraction minus 0.4. Thus, for example, the CpG mutation rate is about 25% higher than average with 30% local GC content and about 50% lower than average with 60% local GC content. We also found empirically from comparing test regions with different CpG and overall GC site frequencies that the coefficient of Δ_{GC} appeared to be approximately 2, in good agreement with the published results. For our final correction, we used a value of 2.5 ± 1.0 for this coefficient.

Mutation rate heterogeneity

In much of our theoretical and methodological discussion, we have assumed that μ is a constant parameter, but in fact different portions of the genome can have different local mutation rates (see Discussion). To learn about the effects that regional variability in μ might have on $H_S(d)$, we use simulations in which we create data with variability in the mutation rate throughout the genome. Our goal is to approximate the true level of variability present in our data on length scales of several tens of kb, which we do by considering polymorphism rates from populations that are distantly related to those we are using to compute $H_S(d)$. Specifically, for each super-region sequence (i.e., each independent segment, including flanking regions; see Methods) being simulated, we divide the length into even thirds and assign a mutation rate for each third proportional to the frequency of doubleton sites (those with exactly two sampled non-reference alleles) in that interval among eight full African genomes (two each San, Dinka, Mbuti, and Yoruba) [24, 28]. The exact rates are chosen in such a way that the overall average rate over the entire genome is equal to a specified value (e.g., 2.5×10^{-8}). When calling a doubleton, we require at most one individual to be masked (see Methods), and if there are fewer than 10,000 un-masked sites in any window, we assign the genome-wide average

rate for that third of the super-region instead.

We note that while this diversity metric does not precisely capture the true variation in mutation rates across the genome, it should be sufficient for our purposes. The frequency of doubleton sites in the African individuals depends on the local coalescent trees, although this particular statistic should be relatively stable (as opposed to, for example, the total number of polymorphic sites) and will also be smoothed somewhat by considering windows typically on the order of 100 kb. Second, given a local tree, there is additional Poisson noise in the observed counts of doubleton mutations. However, because we are using simulations to gauge the effect of rate variability rather than attempting to add matching variability to the calibration data, we do not need the local estimates to be exact. This is especially true because both sources of stochasticity should cause the apparent variability to be too high rather than too low. Finally, the simulation approach avoids the issue of potential correlations between the African diversity and our non-African data as a result of deep shared ancestry.

Additionally, we note that our estimates should not be impacted by multiple-nucleotide mutation events. It has been estimated that up to a few percent of human point mutations involve nearby clustered nucleotide changes [26,36,47], meaning that our inferred rate will correspond to the number of single-base changes per base per generation, rather than the number of independent mutational events per base per generation. However, this is simply a question of how the mutation rate is defined (and moreover, the two are very similar), and all previous mutation rate estimates, to our knowledge, also use the first definition.

Population heterogeneity and admixture

Our basic model assumes that all of the genomes used to calculate $H_S(d)$ are drawn from the same population. Thus, to the extent that our set of individuals are from groups with

different historical sizes, the real data could have different TMRCA patterns in different genomes, whereas the calibration data will be based on the population size profile inferred from the aggregate of all of the samples. A similar phenomenon occurs on a within-genome scale if the test genomes are admixed, in which case individual chromosomes consist of alternating blocks derived from each ancestral mixing population. Although we avoid populations with substantial recent admixture, almost all human populations have experienced some degree of admixture at some point in their history.

Separately, our estimates could potentially be affected by inter-population differences in fine-scale recombination rates. However, the "shared" version of the AA map is intended to apply broadly to non-Africans [17], and it is reasonable to expect that recombination maps will be similar among non-African populations based on their relatively limited diversity of PRDM9 alleles [17, 48, 49].

As with mutation rate heterogeneity, it is difficult to quantify the exact divergence and admixture parameters for the populations under consideration, and hence we use empirical and simulation approaches to study their effects on our inferences. First, while our primary data set consists of a combination of European and East Asian individuals (see Results), we also apply our analyses to the continental groups separately. This allows us both to compare results for populations with different admixture histories and also to compare more homogeneous data sets to the full set with individuals combined from diverged populations.

Second, we perform simulations in which we apply our method to an admixed population designed to emulate present-day Europeans [33, 50]. We simulate a population that experienced a 50/50 mixture 200 generations ago between two populations that had been diverged for 1000 generations. The population sizes are 10,000 in the shared ancestral population before a bottleneck beginning 2000 generations ago; then 1000 during the

bottleneck, which continued until 1000 generations ago (so that the last 200 generations are separate in the two mixing populations); then 15,000 and 7500 in the two mixing populations after the bottleneck; and finally 25,000 after the admixture.

Natural selection

A similar issue to within-genome variation in demography and in the mutation rate is that of heterogeneity in selective effects. Until now we have assumed implicitly that all loci are neutral with respect to fitness, and thus their TMRCAs follow the distributions implied by the standard coalescent model. However, if some sites have non-zero effects on fitness, then two phenomena can result. First, there can be direct selection on new mutations (and also GC-biased gene conversion, which acts similarly to natural selection [21, 44, 51, 52]; see Methods), causing their frequencies to increase or decrease more than expected due to drift. This effect should be minor for our data, since we are considering mutational patterns over relatively short time scales, and if anything, it would likely cause us to underestimate the true mutation rate (see Discussion). The second phenomenon is linked or background selection, whereby local genealogies near selected sites will have different properties from the genome-wide average. In this case, our calibration data, which are based on the average TMRCA distribution, will differ from the real data, but because our PSMC inferences still capture the true genome-wide ancestral population size history, the local variation in this profile will likely only be of second-order importance. Moreover, the effects of linked selection should be similar to those of rate and demographic heterogeneity, since different local genealogical properties caused by selection are analogous to different local mutation rates and/or different local ancestry. Thus, because all three phenomena lead to changes in local levels of diversity, any overall impact of natural selection should be captured in our rate-heterogeneity and admixture simulations described above.

Symmetry of uncertainty

An implicit assumption we make in computing the uncertainty associated with our estimates is that this uncertainty is symmetric above and below the mean. To test this assumption, we used our results for simulated data. For each of our seven simulation scenarios, we rescaled the 25 independent estimates of μ to have mean 0 and standard deviation 1, after which we pooled all 175 data points. The resulting set has a slight positive skewness of 0.14 (see S5 Fig), but this is not statistically significantly different from 0, as the standard deviation of the distribution of sample skewness for 175 observations from a standard normal is approximately 0.18. Thus, we do not see any strong evidence that our uncertainty is greater either above or below the mean.

References

44. Schaibley VM, Zawistowski M, Wegmann D, Ehm MG, Nelson MR, et al. (2013) The influence of genomic context on mutation patterns in the human genome inferred from rare variants. Genome Res 23: 1974–1984.

45. Pratto F, Brick K, Khil P, Smagulova F, Petukhova GV, et al. (2014) Recombination initiation maps of individual human genomes. Science 346: 1256442.

46. Fryxell KJ, Moon WJ (2005) CpG mutation rates in the human genome are highly dependent on local GC content. Mol Biol Evol 22: 650–658.

47. Harris K, Nielsen R (2014) Error-prone polymerase activity causes multinucleotide mutations in humans. Genome Res 24: 1445–1454.

48. Baudat F, Buard J, Grey C, Fledel-Alon A, Ober C, et al. (2010) PRDM9 is a major determinant of meiotic recombination hotspots in humans and mice. Science 327: 836–840.

49. Berg IL, Neumann R, Lam KWG, Sarbajna S, Odenthal-Hesse L, et al. (2010) PRDM9 variation strongly influences recombination hot-spot activity and meiotic instability in humans. Nat Genet 42: 859–863.

50. Lazaridis I, Patterson N, Mittnik A, Renaud G, Mallick S, et al. (2014) Ancient human genomes suggest three ancestral populations for present-day Europeans. Nature 513: 409–413.

51. Capra JA, Hubisz MJ, Kostka D, Pollard KS, Siepel A (2013) A model-based analysis of GC-biased gene conversion in the human and chimpanzee genomes. PLoS Genet 9: e1003684.

52. Lachance J, Tishkoff SA (2014) Biased gene conversion skews allele frequencies in human populations, increasing the disease burden of recessive alleles. Am J Hum Genet 95: 408–420.
