# Supplementary material for: Calibrating the Human Mutation Rate via Ancestral Recombination Density in Diploid Genomes
Source: PLoS Genet. 2015 Nov 12;11(11):e1005550. doi: 10.1371/journal.pgen.1005550 (PMC4642934; doi:10.1371/journal.pgen.1005550)
Supplement: S1 Fig — Blue shading: included in jackknife procedure; red shading: included in final standard error; cross-hatched shading: uncertainty partially integrated into jackknife and partially included separately in final standard error; bold: tested with simulations; italic: tested empirically with real data. We note that while demographic uncertainty is not explicitly included, we show via simulation that this does not cause our standard error to be underestimated. (PDF) [file pgen.1005550.s002.pdf]

Effect	Figure references
**Finite sample size**	**2A, 2B**
**Genetic map error**	**2A, 2B**
**Coalescent simulation**	**2A, 2B**
Interpolation and least-squares fitting	***2A, 2B***
**Demographic parameter estimation**	**2A, 2B**
Uncertainty in α	S2
**Genotype error**	**2D, 2G**
**Gene conversion**	**2C**
Base content and mutability	
**Within-genome rate heterogeneity**	**2E, 2G**
Admixture (population heterogeneity)	***2F, 2G, 3A-C***

Figure S1. Guide to potential sources of uncertainty associated with our method. Blue shading: included in jackknife procedure; red shading: included in final standard error; cross-hatched shading: uncertainty partially integrated into jackknife and partially included separately in final standard error; bold: tested with simulations; italic: tested empirically with real data. We note that while demographic uncertainty is not explicitly included, we show via simulation that this does not cause our standard error to be underestimated.
