# Supplementary material for: Calibrating the Human Mutation Rate via Ancestral Recombination Density in Diploid Genomes
Source: PLoS Genet. 2015 Nov 12;11(11):e1005550. doi: 10.1371/journal.pgen.1005550 (PMC4642934; doi:10.1371/journal.pgen.1005550)
Supplement: S2 Fig — All estimates use our standard data set of eight non-African genomes. Data points represent the inferred rates (independent point estimates), and the lines are linear regression fits for each of the three choices of S as a function of α. We caution that the values for S = 1–5 and 10–20 are less confident than those for our standard range of 5–10. In particular, we believe that the genotype error correction is likely too strong for S = 1–5, and thus the 1–5 values here are too low, but we do not have sufficient statistical power to generate a separate error estimate. We also note that the dependence of μ on α is stronger for larger S, because a higher heterozygosity at the starting points leads to a steeper relaxation of H S(d), so that the curve is more sensitive to the smoothing caused by map error. (PDF) [file pgen.1005550.s003.pdf]

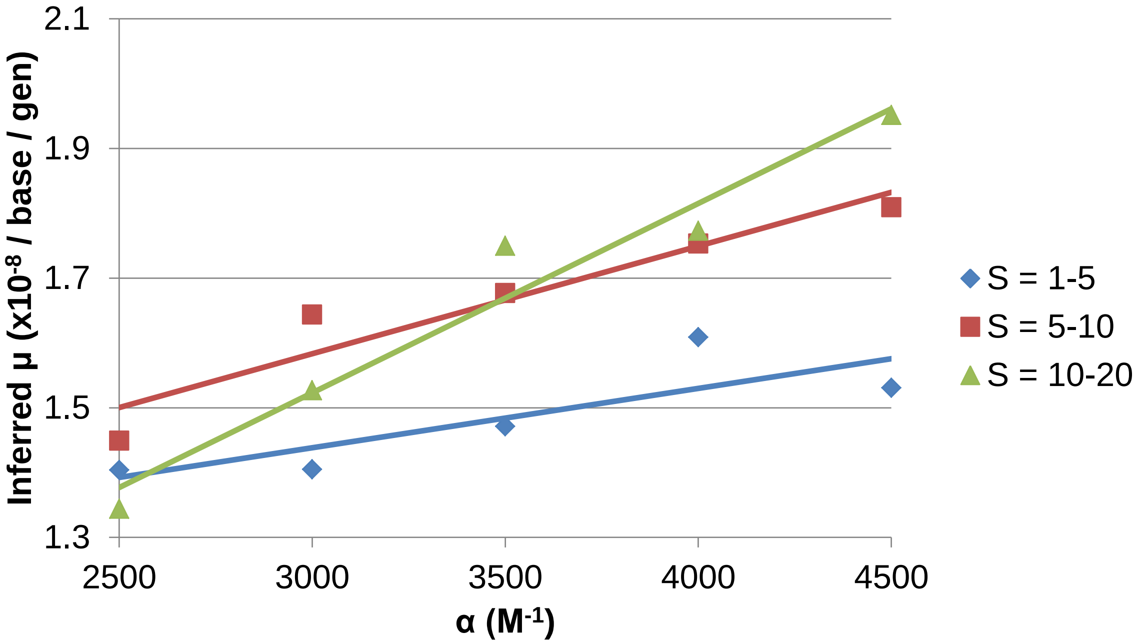

Figure S2. Inferred mutation rates for a range of values of the genetic map error parameter α and starting heterozygosity range S. All estimates use our standard data set of eight non-African genomes. Data points represent the inferred rates (independent point estimates), and the lines are linear regression fits for each of the three choices of S as a function of α. We caution that the values for $S = 1$–5 and 10–20 are less confident than those for our standard range of 5–10. In particular, we believe that the genotype error correction is likely too strong for $S = 1$–5, and thus the 1–5 values here are too low, but we do not have sufficient statistical power to generate a separate error estimate. We also note that the dependence of μ on α is stronger for larger S, because a higher heterozygosity at the starting points leads to a steeper relaxation of $H_S(d)$, so that the curve is more sensitive to the smoothing caused by map error.
