# Supplementary material for: Calibrating the Human Mutation Rate via Ancestral Recombination Density in Diploid Genomes
Source: PLoS Genet. 2015 Nov 12;11(11):e1005550. doi: 10.1371/journal.pgen.1005550 (PMC4642934; doi:10.1371/journal.pgen.1005550)
Supplement: S3 Fig — (A) CpGs only; the inferred rate is μ = 0.50 ± 0.06 × 10^−8^. (B) Non-CpGs only; the inferred rate is μ = 1.36 ± 0.13 × 10^−8^. (PDF) [file pgen.1005550.s004.pdf]

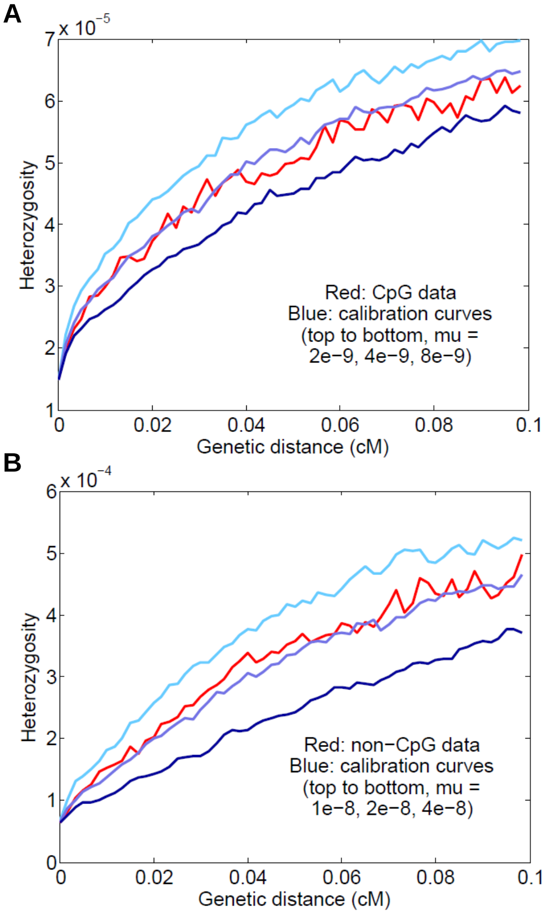

Figure S3. Results for CpG transitions and all other mutations separately, using our primary eight-genome data set. (A) CpGs only; the inferred rate is $\mu = 0.50 \pm 0.06 \times 10^{-8}$. (B) Non-CpGs only; the inferred rate is $\mu = 1.36 \pm 0.13 \times 10^{-8}$.
