# Supplementary material for: Calibrating the Human Mutation Rate via Ancestral Recombination Density in Diploid Genomes
Source: PLoS Genet. 2015 Nov 12;11(11):e1005550. doi: 10.1371/journal.pgen.1005550 (PMC4642934; doi:10.1371/journal.pgen.1005550)
Supplement: S4 Fig — (A) Simulated data: we create test data using the prior but omit it for the calibration data. The curve shapes are markedly different, as the calibration curves relax too slowly at the smallest values of d. It is also apparent that the inferred value of μ is lower than the true value of 2.5 × 10^−8^. (B) Real data for eight non-African genomes. We observe a very similar discrepancy between the real-data and calibration curves (compare Fig 4A). (PDF) [file pgen.1005550.s005.pdf]

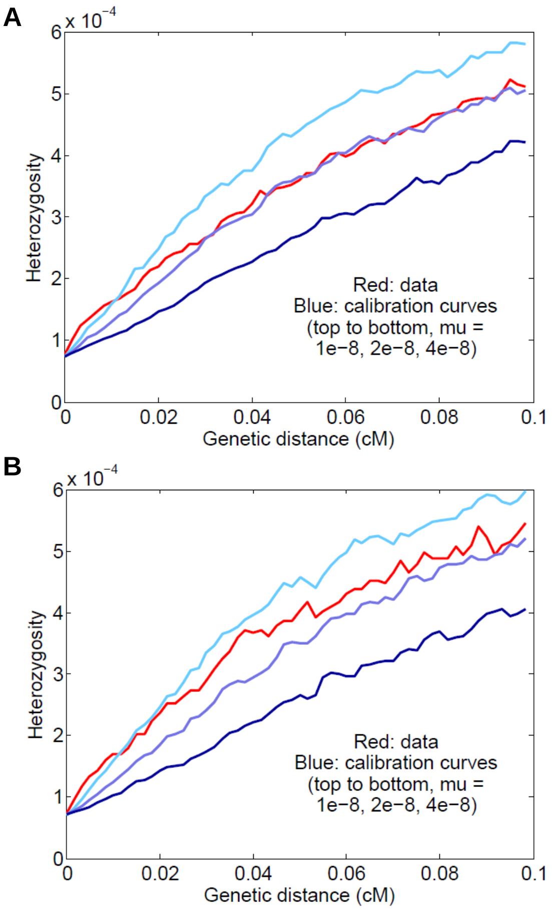

Figure S4. $H_{5\text{–}10}(d)$ curves without the pseudo-count prior. (A) Simulated data: we create test data using the prior but omit it for the calibration data. The curve shapes are markedly different, as the calibration curves relax too slowly at the smallest values of d. It is also apparent that the inferred value of μ is lower than the true value of 2.5×10^{-8}. (B) Real data for eight non-African genomes. We observe a very similar discrepancy between the real-data and calibration curves (compare Figure 4A).
