# Supplementary material for: Calibrating the Human Mutation Rate via Ancestral Recombination Density in Diploid Genomes
Source: PLoS Genet. 2015 Nov 12;11(11):e1005550. doi: 10.1371/journal.pgen.1005550 (PMC4642934; doi:10.1371/journal.pgen.1005550)
Supplement: S5 Fig — We standardized the 25 independent estimates of μ for each of the seven simulated scenarios and combined all 175 values to test for skewness (see S1 Text). (PDF) [file pgen.1005550.s006.pdf]

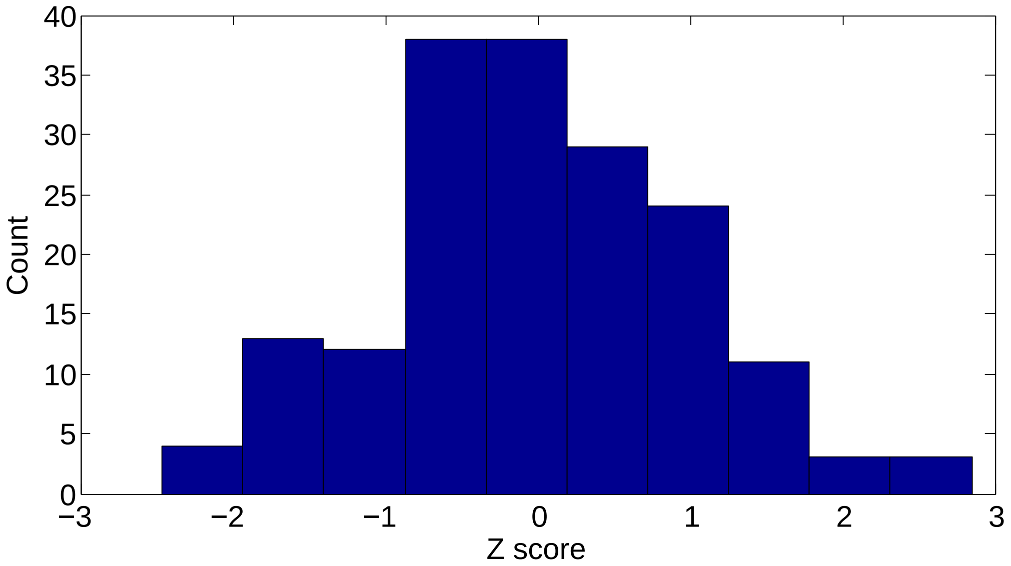

Figure S5. Histogram of Z scores of simulation results. We standardized the 25 independent estimates of μ for each of the seven simulated scenarios and combined all 175 values to test for skewness (see Text S1).
