# Supplementary material for: Calibrating the Human Mutation Rate via Ancestral Recombination Density in Diploid Genomes
Source: PLoS Genet. 2015 Nov 12;11(11):e1005550. doi: 10.1371/journal.pgen.1005550 (PMC4642934; doi:10.1371/journal.pgen.1005550)
Supplement: S1 Table — Sequence comparisons, with implied divergence times (mean ± standard deviation, in millions of years, using our inferred mutation rate of 1.61 ± 0.13 × 10^−8^ per base per generation and an average generation interval of 29 years), for sites in the genome passing filters. The first 14 lines represent divergence between the two chromosomes within the individual genomes in our data set (suffix “A” from [24] and suffix “B” from [28], except both Australians from the latter), based on genome-specific filtering (see Methods). Human–chimpanzee statistics are averaged over the filters for the first eight genomes; we note that the third column represents the TMRCA of the two species’ reference sequences rather than the population split time (see Discussion). (PDF) [file pgen.1005550.s007.pdf]

Table S1. Sequence divergence for sites passing filters

Sequence comparison	Per-base diff	Divergence time (My)
French A	7.41×10^{-4}	0.67 ± 0.06
French B	7.45×10^{-4}	0.67 ± 0.06
Sardinian A	7.33×10^{-4}	0.66 ± 0.06
Sardinian B	7.23×10^{-4}	0.65 ± 0.05
Han A	7.05×10^{-4}	0.64 ± 0.05
Han B	7.00×10^{-4}	0.63 ± 0.05
Dai A	7.05×10^{-4}	0.63 ± 0.05
Dai B	7.00×10^{-4}	0.63 ± 0.05
Australian A	6.47×10^{-4}	0.58 ± 0.05
Australian B	6.53×10^{-4}	0.59 ± 0.05
Karitiana A	5.35×10^{-4}	0.48 ± 0.04
Karitiana B	5.39×10^{-4}	0.49 ± 0.04
Papuan A	5.95×10^{-4}	0.54 ± 0.05
Papuan B	5.86×10^{-4}	0.53 ± 0.04
Human–chimpanzee	1.23×10^{-2}	11.1 ± 0.9

Sequence comparisons, with implied divergence times (mean ± standard deviation, in millions of years, using our inferred mutation rate of $1.61 \pm 0.13 \times 10^{-8}$ per base per generation and an average generation interval of 29 years), for sites in the genome passing filters. The first 14 lines represent divergence between the two chromosomes within the individual genomes in our data set (suffix "A" from [23] and suffix "B" from [27], except both Australians from the latter), based on genome-specific filtering (see Methods). Human–chimpanzee statistics are averaged over the filters for the first eight genomes; we note that the third column represents the TMRCA of the two species' reference sequences rather than the population split time (see Discussion).
