# Supplementary material for: Calibrating the Human Mutation Rate via Ancestral Recombination Density in Diploid Genomes
Source: PLoS Genet. 2015 Nov 12;11(11):e1005550. doi: 10.1371/journal.pgen.1005550 (PMC4642934; doi:10.1371/journal.pgen.1005550)
Supplement: S2 Table — Statistics for the ascertained regions in our samples: the primary set of eight genomes (two each French, Sardinian, Han, and Dai), the four Europeans alone, the four East Asians alone, and two each Australian, Karitiana, and Papunan. The regions are selected as having 5–10 heterozygous sites per 100 kb (after filtering) in 100 kb windows for Europeans and East Asians and 5–10 heterozygous sites per 100 kb for the other three populations. RR = recombination rate (cM/Mb), measured in different-sized windows around the starting points (e.g., 10 kb refers to the 10 kb window centered at the midpoint of the ascertained region; s.r. = full super-regions). CpG site fraction and GC content fraction are measured in 30 kb windows (see Methods). (PDF) [file pgen.1005550.s008.pdf]

Table S2. Descriptive statistics for ascertained genomic regions

	**All 8**	**Europe**	**East Asia**	**Australian**	**Karitiana**	**Papuan**
# windows	9626	4441	5185	6368	4765	6451
$H_S(0)$	7.44E-05	7.28E-05	7.57E-05	7.12E-05	6.54E-05	7.11E-05
RR (10 kb)	0.53	0.50	0.55	0.59	0.68	0.65
RR (100 kb)	0.58	0.58	0.59	0.66	0.75	0.70
RR (s.r.)	0.66	0.66	0.66	0.69	0.74	0.71
CpG sites	0.0130	0.0129	0.0132	0.0132	0.0132	0.0134
GC content	0.38	0.38	0.39	0.38	0.39	0.39

Statistics for the ascertained regions in our samples: the primary set of eight genomes (two each French, Sardinian, Han, and Dai), the four Europeans alone, the four East Asians alone, and two each Australian, Karitiana, and Papuan. The regions are selected as having 5–10 heterozygous sites per 100 kb (after filtering) in 100 kb windows for Europeans and East Asians and 1–15 heterozygous sites per 100 kb for the other three populations. RR = recombination rate (cM/Mb), measured in different-sized windows around the starting points (e.g., 10 kb refers to the 10 kb window centered at the midpoint of the ascertained region; s.r. = full super-regions). CpG site fraction and GC content fraction are measured in 30 kb windows (see Methods).
